# Morphological study of TNPO3 and SRSF1 interaction during myogenesis by combining confocal, structured illumination and electron microscopy analysis

**DOI:** 10.1007/s11010-020-04023-y

**Published:** 2021-01-15

**Authors:** Roberta Costa, Maria Teresa Rodia, Nicoletta Zini, Valentina Pegoraro, Roberta Marozzo, Cristina Capanni, Corrado Angelini, Giovanna Lattanzi, Spartaco Santi, Giovanna Cenacchi

**Affiliations:** 1 Department of Biomedical and Neuromotor Sciences—DIBINEM, Alma Mater Studiorum University of Bologna, via Massarenti 9, 40138 Bologna, Italy; 2 Center of Applied Biomedical Research—CRBA, Alma Mater Studiorum University of Bologna, St. Orsola Hospital, via Massarenti 9, 40138 Bologna, Italy; 3 CNR—National Research Council of Italy, Institute of Molecular Genetics “Luigi Luca Cavalli-Sforza”, Unit of Bologna, via di Barbiano 1/10, 40136 Bologna, Italy; 4 IRCCS Istituto Ortopedico Rizzoli, via di Barbiano 1/10, 40136 Bologna, Italy; 5 Neuromuscular Unit, Neurobiology Research group, IRCCS San Camillo Hospital, via Alberoni 70, 30126 Venice, Italy

**Keywords:** TNPO3, SRSF1, Myogenesis, Structured illumination microscopy, Electron microscopy

## Abstract

Transportin3 (TNPO3) shuttles the SR proteins from the cytoplasm to the nucleus. The SR family includes essential splicing factors, such as SRSF1, that influence alternative splicing, controlling protein diversity in muscle and satellite cell differentiation. Given the importance of alternative splicing in the myogenic process and in the maintenance of healthy muscle, alterations in the splicing mechanism might contribute to the development of muscle disorders. Combining confocal, structured illumination and electron microscopy, we investigated the expression of TNPO3 and SRSF1 during myogenesis, looking at nuclear and cytoplasmic compartments. We investigated TNPO3 and its interaction with SRSF1 and we observed that SRSF1 remained mainly localized in the nucleus, while TNPO3 decreased in the cytoplasm and was strongly clustered in the nuclei of differentiated myotubes. In conclusion, combining different imaging techniques led us to describe the behavior of TNPO3 and SRSF1 during myogenesis, showing that their dynamics follow the myogenic process and could influence the proteomic network necessary during myogenesis. The combination of different high-, super- and ultra-resolution imaging techniques led us to describe the behavior of TNPO3 and its interaction with SRSF1, looking at nuclear and cytoplasmic compartments. These observations represent a first step in understanding the role of TNPO3 and SRFSF1 in complex mechanisms, such as myogenesis.

## Introduction

Transportin 3 (TNPO3) is a karyopherin β that works as a nuclear carrier shuttling, from the cytoplasm to the nucleus, the serine/arginine-rich proteins (SR proteins) [1]. The SR protein family includes 12 members, each comprising one or more RNA-recognition motifs (RRM) and a nuclear localization signal (NLS) made of a sequence rich in Arg-Ser, the SR domain [2]. TNPO3 is composed of 20 consecutive hairpin motifs, or HEAT repeats [3], that create a structure with high plasticity responsible for the ability to bind different proteins [4] and that gives to TNPO3 a toroidal shape with N- and C-terminal regions facing each other [1]. Generally, the N-terminal binds RanGTP, whereas the C-terminal carries the cargo [5]. TNPO3 works as carrier following the rules of protein trafficking in eukaryotic cells, recognizing specific import signals within its cargoes [6–10]. The SR family includes essential splicing factors and proteins involved in mRNA splicing and metabolism [11]; they play key role in pre-mRNA splicing, in selecting alternative splice site [12] and they participate in transcription regulation, mRNA transport, translation and nonsense mRNA decay [2]. Some SR proteins, such as the splicing factors SRSF1 (or SF2/ASF), and SRSF2 (or SC35) and CPSF6 (cleavage and polyadenylation-specific factor 6) have been described as specific cargoes of TNPO3 [1]. Although all the SR proteins are predominantly nuclear and localize to interchromatin granule clusters or nuclear speckles, six of them (SRSF1, SRSF3, SRSF4, SRSF6, SRSF7, SRSF10) can shuttle between the nucleus and the cytoplasm [13–15]. The SR proteins that work as essential splicing factors influence the post transcriptional gene regulation, affecting the proteomic diversity in muscle, and contribute to the control of satellite cell fate during muscle differentiation, helping the formation and maintenance of healthy skeletal muscle [16–18]. Interestingly, some mutations causing alteration in splicing are responsible for abnormalities in muscle fibers and contribute to muscle diseases [18, 19]. In this study we analyzed the possibility that the fine tuning of myogenic differentiation could be modulated by interactions between the splicing factor SRSF1 and its carrier TNPO3. We monitored the different steps of myogenesis in C2C12, murine myoblasts which derive from satellite cells and represent a good model to recapitulate myogenic differentiation. In detail we investigated early, intermediate and late stage of differentiation; the early stage (at 24 h of differentiation) is not characterized by clear morphological changes, while the intermediate stage (3–5 days of differentiation) is characterized by the presence of some myotubes containing more than two nuclei and in the late step (10 days of differentiation) the presence of long multinucleated myotubes overpass the underlying mononucleated myoblasts [20]. Besides the morphological analyses we investigated the different steps of myogenesis through quantitative analyses of specific differentiation markers [21]. TNPO3 expression has been investigated by confocal and electron microscopy during myogenesis and the variations of TNPO3 and SRSF1 have been quantitatively evaluated in the cytoplasmic and nuclear compartments through advanced imaging systems. The results obtained stress the role of TNPO3 as carrier of SRSF1 in crucial steps of the myogenesis and could shed light on their fine interaction during myoblast differentiation.

## Materials and methods

### Cell cultures and myogenic differentiation

The murine myoblasts C2C12 (ATCC Cat# CRL-1772, RRID:CVCL_0188) were grown in complete culture medium at 37 °C, 5% CO2. At 80% confluence, C2C12 were induced to differentiate replacing complete culture medium with a differentiation medium and myogenic differentiation was investigated at the following stages: T0, proliferating undifferentiated myoblast used as control; T1, early stage at 24 h of differentiation; T3–T5, intermediate stage after 3–5 days of differentiation; T10, late stage, myotubes after 10 days of differentiation. Media composition in Table 1.

**Table 1:** Composition of cell culture media

Type of medium	Composition	Company
Growth medium	DMEM (Dulbecco’s Modified Eagle Medium)	Biowest, Nuaille, France
	1% L-Glutamine	Euroclone, Milan, Italy
	1% penicillin/streptomycin	
	10% heat inactivated fetal bovine serum (FBS)	
Differentiation medium	DMEM (Dulbecco’s Modified Eagle Medium)	Biowest, Nuaille, France
	1% L-Glutamine	Euroclone, Milan, Italy
	1% penicillin/streptomycin	
	1% of heat inactivated equine serum (HS)	Sigma-Aldrich, St.Louis, Missouri, USA

### RNA isolation and qRT-PCR

RNA from C2C12 was extracted using TRIZOL® Reagent (Thermo Fischer Scientific, Waltham, Massachusetts, USA) and chloroform/isopropanol purification method. Total RNA quantity and quality were determined using NanoDrop ND-2000 (Thermo Fischer Scientific). One microgram of RNA was reverse transcribed with RevertAid First Strand cDNA Synthesis kit and Real-time qPCR was performed with MaximaTM SYBR Green qPCR Master Mix 2X (both kits from Thermo Fischer Scientific) in Thermal Cycler RT-PCR Detection System IQ5 (BioRad, Hercules, California, USA). Reaction efficiency (E) was calculated as previously described [22]. Real-time qPCR analysis was performed in triplicate and qPCR signals (CT) were normalized to glyceraldehyde 3-phosphate dehydrogenase (GAPDH) for C2C12. Primers list in Table 2.

**Table 2:** List of genes and primer sequences used for RT-PCR

Genes	NCBI RefSeq	Primer pairs sequence (5′->3′)	RT-PCR product size (base pair)
Tnpo3	NM_177296.4	GAGTTTCGAATGAGAGTGTC CAGCCATGATAAAGAGAACC	145
MyoD	NM_010866.2	GCTTAAATGACACTCTTCCC AGGACTACAACAACAACAAC	131
Myf5	NM_008656.5	AGGTGGAGAACTATTACAGC TGATACATCAGGACAGTAGATG	152
Desmin	NM_010043.2	ACACCTAAAGGATGAGATGG GAGAAGGTCTGGATAGGAAG	147
Pax7	AF254422.4	GTATAAGAGAGAGAACCCCG GCCATCTTCTTCTTTCTTGTC	175
MyoG	NM_031189.2	AGTACATTGAGCGCCTAC CAAATGATCTCCTGGGTTG	182
Myf6	NM_008657.2	ATAACTGCTAAGGAAGGAGG AAGAATGTTCCAAATGCTGG	160
GAPDH	NM_001256799.2	CTCTGATTTGGTCGTATTGG GTAAACCATGTAGTTGAGGTC	111

### MicroRNAs analysis and exosomes isolation

C2C12 for miRNAs analysis were cultured in complete culture medium and at 80% confluence induced to differentiate. At each differentiation stage (T0–T10) cells were recovered by enzymatic digestion and 2.5 × 106 cells stored at −80 °C for subsequent study of miRNAs. For the isolation of exosomes released from C2C12 in the culture medium, the supernatants were recovered, centrifuged (300 *g* for 10 min at 4 °C) and filtered with a 0.2 μm filter. The filtered supernatants were centrifuged with Beckman–Coulter ultracentrifuge at 120,000 *g* for 70 min at 4 °C and the pellets, containing the exosomes, stored at −80 °C. MiR-1, miR-206, miR-133a and 133b were isolated and analyzed as previously described [23].

### Protein extraction from total, cytoplasmic and nuclear fraction

Protein expression in C2C12 has been evaluated in total cell lysate and in nuclear and cytoplasmic fractions. For total cell fraction C2C12 were lysed with RIPA buffer plus Protease Inhibitors Cocktail (Sigma-Aldrich, Missouri, USA) and Na3Va4; after 30 min on ice and centrifugation (20 min at 12,800 *g*), supernatants were collected and stored at −80 °C.

For protein extraction from nuclear and cytoplasmic fractions, 1–5 × 106 cells were pelleted, lysed in five volumes of hypotonic lysis buffer and centrifuged 5 min at 1,850 *g*. The cell lysate was then incubated with two volumes of hypotonic buffer for 10 min on ice and centrifuged (1,850 *g* for 15 min at 4 °C) to separate the nuclear fraction (in the pellet) and the cytoplasmic fraction (in the supernatant). The cytoplasmic fraction was added with 0.11 volumes of S100 buffer and centrifuged at 40,000 *g* for 30 min; the supernatant containing the cytoplasmic protein was stored at −80 °C. Nuclear fraction was rinsed with half and half volume of low salt and high salt buffer respectively and incubated on ice (30 min). The nuclear protein suspension was centrifuged (13,225 *g* for 30 min at 4 °C) and the supernatant stored to −80 °C. Protein concentration was determined by DC Protein Assay (BioRad) using BSA as standard. Buffers composition in Table 3.

**Table 3:** Composition of buffers used for protein extraction from nuclear and cytoplasmic fractions

Type of buffer	Composition	Company
RIPA	25 mM Tris-HCl pH 7.5	Sigma-Aldrich, St.Louis, Missouri, USA
	50 mM NaCl_2_	
	0.5% Na-deoxycholate	Thermo Fisher Scientific, Waltham, Massachusetts, USA
	1% NP-40	Sigma-Aldrich, St.Louis, Missouri, USA
	0.1% SDS	
Hypotonic lysis buffer	10 mM HEPES pH 7.9	All from Sigma-Aldrich, St.Louis, Missouri, USA Missouri, USA
	1.5 mM MgCl2	
	10 mM KCl	
	complete^TM^ EDTA-Free 2×	Roche, Basle, Switzerland
S100 buffer	0.3 M HEPES pH 7.9	All from Sigma-Aldrich, St.Louis, Missouri, USA
	30 mM MgCl_2_	
	1.4 mM KCl	
	complete^TM^ EDTA-Free 2×	Roche, Basle, Switzerland
Low salt buffer	20 mM HEPES pH 7.9	Sigma-Aldrich, St.Louis, Missouri, USA
	25% glycerol	Thermo Fisher Scientific, Massachusetts, USA
	20 mM KCl	All from Sigma-Aldrich, St.Louis, Missouri, USA
	1.5 mM MgCl_2_	
	0.2 mM EDTA	
	complete^TM^ EDTA-Free 2×	Roche, Basle, Switzerland
High salt buffer	20 mM HEPES pH 7.9	Sigma-Aldrich, St.Louis, Missouri, USA
	25% glycerol	Thermo Fisher Scientific, Massachusetts, USA
	1.2 M KCl	Sigma-Aldrich, St.Louis, Missouri, USA
	1.5 mM MgCl2	
	complete^TM^ EDTA-Free 2×	Roche, Basle, Switzerland

### Western blotting (WB)

Proteins (40 μg of lysate) were separated on 10% polyacrylamide gel and transferred to a nitrocellulose membrane for immunoblotting. Blots were blocked for 1 h at room temperature (RT) in TBS-Tween 0.1% plus 5% dry milk (Bio-Rad) and membranes were incubated overnight at 4 °C with primary antibodies diluted in PBS 1% BSA. The immunoprobed membranes were washed with TBS-Tween 0.05% and incubated for 1 h at RT with peroxidase-labeled secondary antibodies. Protein presence was detected by chemiluminescent reaction (Clarity Western ECL Substrate, BioRad). Relative intensity of protein expression was calculated using ImageJ and normalized to actin; statistics were performed using T test. Antibodies list in Table 4.

**Table 4:** List of primary and secondary antibodies and dilutions used for WB, IF and IEM analyses

	Dilution	Company
*Primary antibodies*		
Skeletal Muscle Myosin (F59)	For WB: 1/200 For IF: 1/200	Santa Cruz Biotechnology, Dallas, Texas, USA
Myogenin (5FD)	For WB: 1/200 For IF: 1/200	
MyoD (G-1)	For WB: 1/200	
TNPO3 (ab71388)	For WB: 1/1,000 For IF: 1/200 For IEM: 1/20	Abcam, Cambridge, UK
SRSF1 (96)	For WB: 1/250 For IF: 1/100	Thermo Fisher Scientific, Waltham, Massachusetts, USA
Actin (I-19)	For WB:1/500	Santa Cruz Biotechnology, Dallas, Texas, USA
*Secondary antibodies*		
Goat Anti-Mouse IgG (H + L), DyLight 488	For IF: 1/1,000	Thermo Fisher Scientific, Waltham, Massachusetts, USA
Goat Anti-Rabbit IgG (H + L), DyLight 650	For IF: 1/250	
Amersham ECL Anti-mouse IgG HRP-conjugated	For WB: 1/1,000	GE Healthcare, Chicago, Illinois, USA
Amersham ECL Anti-rabbit IgG HRP-conjugated	For WB: 1/1,000	
Anti-goat IgG HRP-conjugated	For WB: 1/10,000	Jackson ImmunoResearch, Cambridge, UK
Goat anti-rabbit conjugated with 10 nm colloidal gold particles	For IEM 1/20	BBInternational, Cardiff, UK

### Immunofluorescence

For immunofluorescence (IF), 1 × 104 cells/cm^2^ were seeded on Nunc LabTek Chamber Slides (Thermo Fisher Scientific) and IF have been performed as previously described [24]. After the incubation with primary antibodies overnight at 4 °C, cells were washed, incubated with secondary antibodies for 1 h at 37 °C and nuclei counterstained with Hoechst (Sigma-Aldrich). Slides were mounted with aqueous medium and different fields for each slide were observed with a fluorescence confocal microscope coupled with a digital camera. Antibodies list in Table 4.

### Confocal imaging and evaluation of TNPO3 fluorescence intensity

Confocal imaging was performed using a Nikon A1 confocal laser scanning microscope, equipped with a 60×, 1.4 NA objective and with 405, 488, and 561 nm laser lines. Z-stacks were collected at optical resolution of 210 nm/pixel, stored at 12-bit with 4096 different gray levels, pinhole diameter set to 1 Airy unit and z-step size to 500 nm. The data acquisition parameters were fixed, such as laser power, gain in amplifier and offset level. All image analyses and 3D rendering were performed using NIS-Elements software (Nikon, RRID:SCR_014329). The degree of fluorescence intensity of TNPO3 can be assessed in a semi-quantitative manner by measure, the mean fluorescence intensity in 50 representative region of interests (ROIs) of nucleus and cytoplasm, through mid-nucleus confocal sections: circular ROI, diameter size of 64 pixels.

### IEM for TNPO3 localization

Cells were fixed in 1% glutaraldehyde in 0.1 M phosphate buffer pH 7.4, for 30 min at RT, scraped-off from petri dishes, pelleted at 1,200 *g* for 20 min and further fixed for 45 min. Pellets were dehydrated in ethanol and embedded in London Resin White at 60 °C. Thin sections were immunolabeled for TNPO3, following a protocol previously described [25]. Controls consisted of samples processed without primary antibody. Thin sections were stained with aqueous uranyl acetate and lead citrate and observed with a Zeiss EM 109 transmission electron microscope. Image were captured using a Nikon digital camera Dmx 1200F and ACT-1 software. No colloidal gold particles were detected in controls (not shown). Antibodies list in Table 4.

### Super resolution microscopy for analysis of SRSF1 and TNPO3 interaction

Super Resolution microscopy (3D N-SIM, Nikon-Structured Illumination Microscopy) was performed using a Plan-Apochromat × 100/1.49 Oil TIRF objective and 405, 488 and 561 nm laser lines. For each axial plane of a 3D stack 1024 × 1024 pixel images and 4096 gray levels were acquired in 3 rotations and 5 different phases. Final images (recorded at z-step size of 125 nm) were reconstructed using NIS-Elements Advanced Research software (Nikon). The colocalization of the fluorochromes was evaluated by comparing the equivalent pixel positions of green and red signals in each of the acquired images (optical sections). A two-dimensional scatter plot diagram of the individual pixels from the paired images was generated and a threshold level of signal to be included in the analysis was selected. Pixels with intensity values greater than 50% grey levels (on a scale from 0 to 4096) were selected for both signals, and the co-localization binary maps that indicate regions containing highly colocalized signals, was imaged and merged (in white) to the green and red signals. The co-localization was quantified using Mander’s Overlap coefficient and expressed as percentage ± SD [26]. Image analysis (volume measurements and 3D object count) was performed using NIS-Elements Advanced Research software.

## Results

### Analysis of TNPO3 expression during myogenic differentiation

**Fig. 1:**
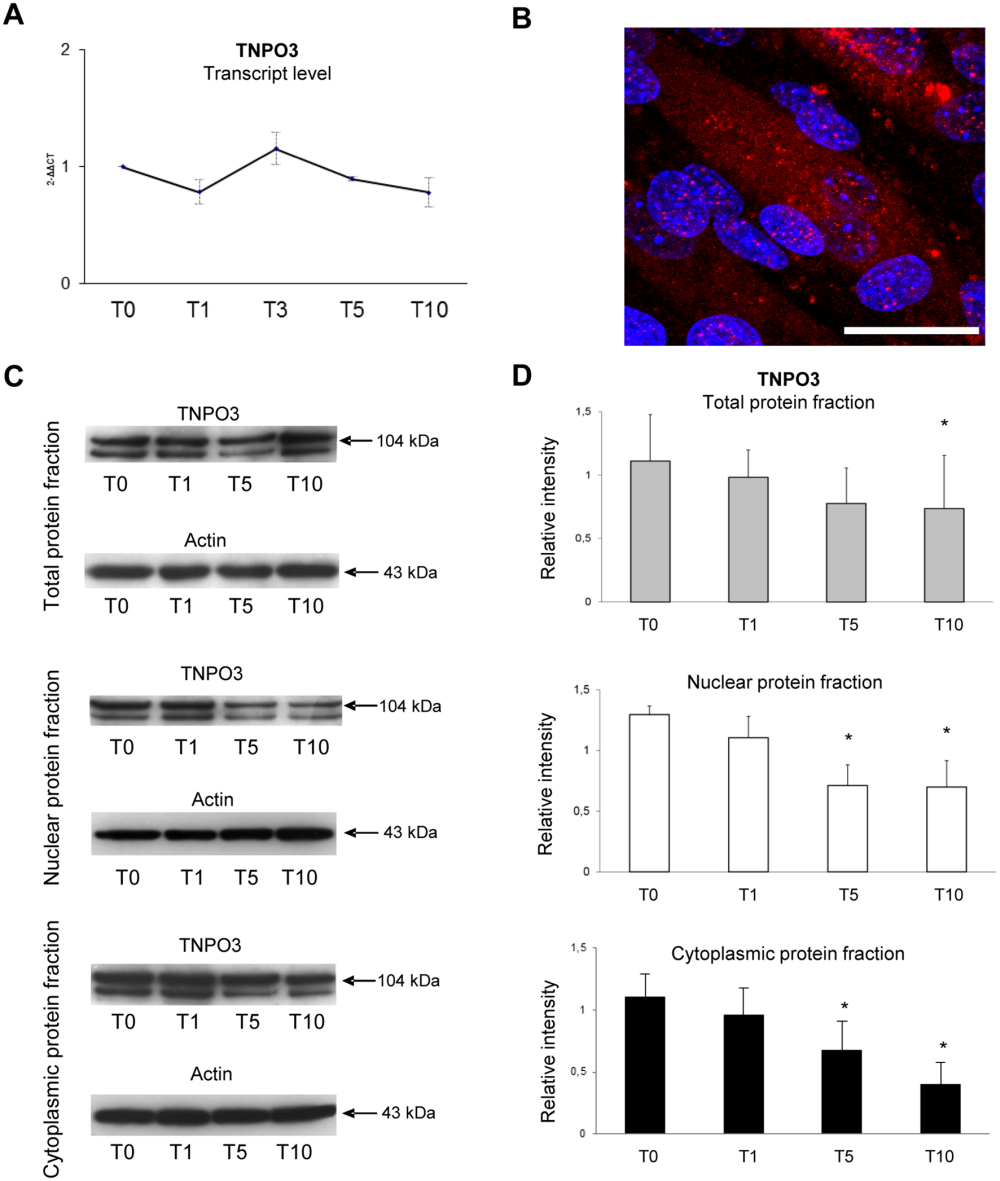
Analysis of TNPO3 expression during myogenesis in C2C12 cells. (**a**) Real-time q-PCR showing TNPO3 transcript level during myogenic differentiation; data are representative of three experiments and expressed as means ± SD. (**b**) IF staining for TNPO3 expression in C2C12 at T10. TNPO3 in red and nuclei in blue (Scale bar: 20 μm). (**c**) Western blotting for TNPO3 in total, nuclear and cytoplasmic protein fractions. The blots show two bands for TNPO3 that are probably due to the presence of different splicing isoforms of TNPO3. (**d**) The bands were quantitated by calculating the relative quantities of TNPO3 normalized to Actin. Data are representative of three experiments and expressed as mean ± SD; the level of significance was set at *p* < 0.05

TNPO3 expression has been investigated during myogenic differentiation allowing us to evaluate its basal expression in undifferentiated myoblasts and along differentiation stages to myotubes formation. Real-time-PCR showed that basal expression of TNPO3 gene decreased at T1, while it returned to basal level with only a slight increase at T3 (Fig. 1a). We also evaluated the protein amount in total, nuclear and cytoplasmic protein fractions. In the cytoplasm TNPO3 decreases with the progression of differentiation: it was present mainly in T0 and it decreased as differentiation proceeded with a significative reduction in T5 and T10. At nuclear level, TNPO3 was highly expressed in T0 and T1, while it is reduced significantly in T5 and T10. The expression of TNPO3 in total protein fraction started to decrease, conform to single nuclear and cytoplasmic fractions, in T5 and T10 (Fig. 1c–d).

**Fig. 2:**
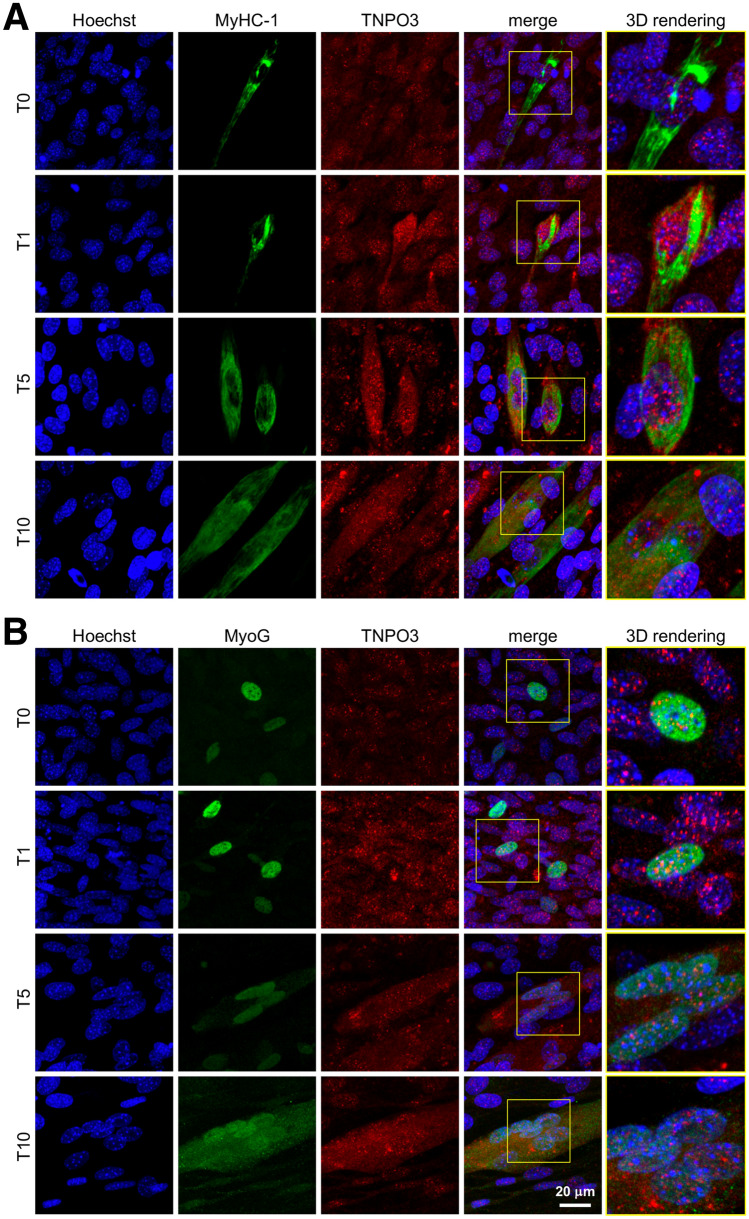
Investigation of TNPO3 localization during myogenesis by confocal microscopy. (**a**) IF double staining for TNPO3 (in red) and MyHC-1 (in green). (**b**) IF double staining for TNPO3 (in red) and MyoG (in green). Nuclei are counterstained with Hoechst (first column, in blue). In the fourth column red and green fluorescent signals are merged and in the fifth column the 3D rendering of the area marked by square. Confocal microscopy investigation showed that during myogenesis TNPO3 tended to increase in intermediate (T5) and late (T10) differentiation steps and it localized mainly in those cells that responded to differentiation stimuli and express MyoG and MyHC-1

In order to determine the influence of myogenic differentiation on the expression and localization of TNPO3, we performed a quantitative confocal microscopy analyses and an ultrastructural immunogold localization of TNPO3 for each type of cell: myoblasts and myotubes. IF observed by confocal microscope highlighted a different localization of TNPO3 during different phases of myogenic differentiation. In undifferentiated myoblasts (T0) TNPO3 was expressed at cytoplasmic and nuclear level and at T1 it increased in both compartments. In T5 we observed a significant decrease in TNPO3 expression in non-differentiating myoblasts, whereas it raised in the nucleus and cytoplasm of fusing myotubes, with a significant increase in T10 (Fig. 1b and 2a–b, red signals). Fluorescence intensity relative to TNPO3 has been quantified distinguishing between undifferentiated myoblasts and differentiated myotubes. We confirmed that fluorescence intensity for TNPO3 decreased in both nuclear and cytoplasmic compartments of undifferentiated myoblasts at T5 and T10, while it increased in differentiated myotubes, particularly in the nucleus (Table 5).

**Table 5:** TNPO3 fluorescence intensity

TNPO3 fluorescence intensity (gray levels ± SD)				
	Undifferentiated myoblasts		Differentiated myotubes	
	(Nucleus)	(Cytoplasm)	(Nucleus)	(Cytoplasm)
T0	354 ± 59	173 ± 81	–	–
T1	525 ± 73	205 ± 54	–	–
T5	38 ± 25	8 ± 7	259 ± 58	201 ± 18
T10	42 ± 38	10 ± 6	452 ± 27	294 ± 60

Fluorescence intensity relative to TNPO3 in both nuclear and cytoplasmic compartments of undifferentiated myoblasts and differentiated myotubes. The fluorescence intensity, as differentiation proceeded, increased in differentiated myotubes, particularly in nuclear domain. Data are representative of three experiments and expressed as mean ± SD

TNPO3 localization has been investigated also in relation to two selected proteins whose expression changes as myogenic differentiation proceeds: myogenin (MyoG) and myosin heavy chain 1 (MyHC-1). Myogenin is directly involved in the entry into myogenic differentiation, while MyHC-I is largely considered a muscle specific protein, since multinucleated myotubes start to express MyHC-I in their developmental sequence toward myofibers [27]. In particular, C2C12 cells have been described to show a progressive increase in MyHC-I expression, starting from the intermediate stage of differentiation and continuing as myogenesis proceeds. We showed that TNPO3 increased and localized mainly in those cells that expressed MyoG and MyHC-1, confirming their commitment to differentiated myotubes (Fig. 2a–b).

**Fig. 3:**
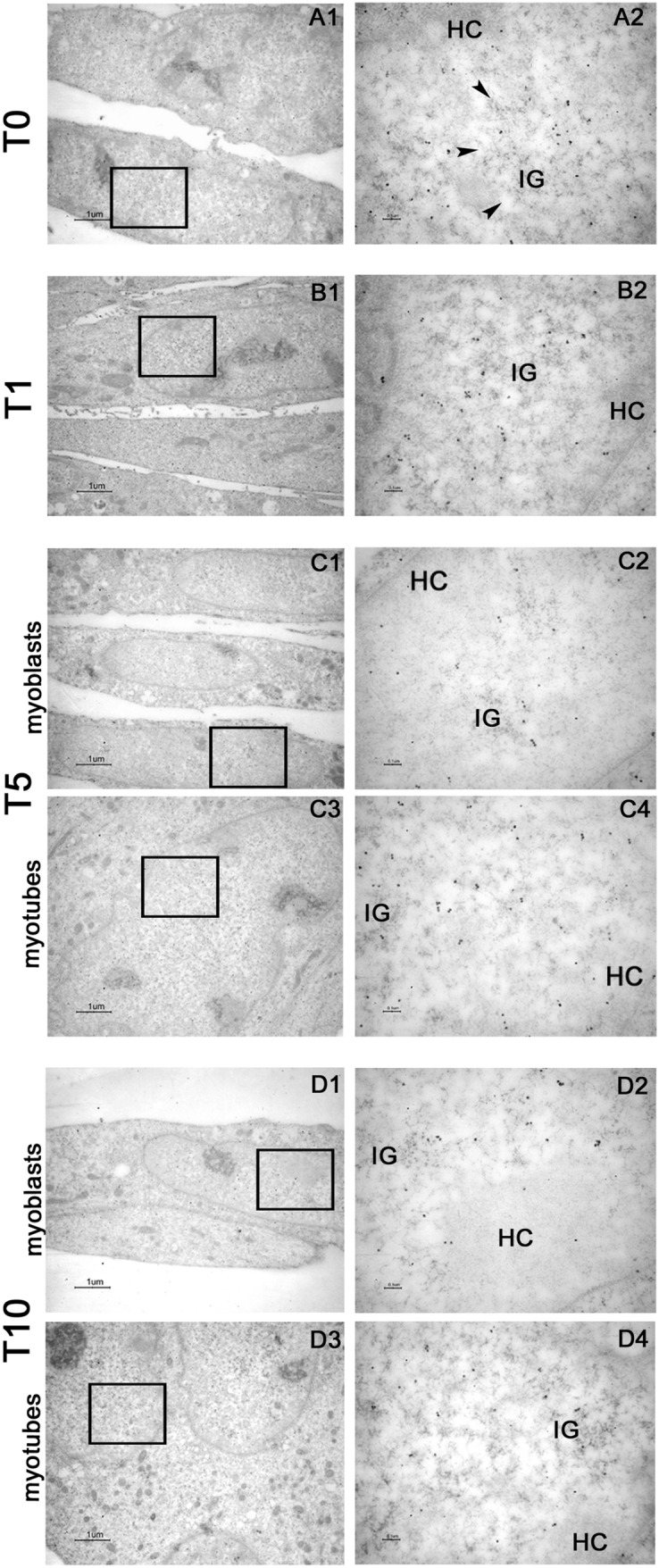
IEM analysis of TNPO3 in C2C12 during myogenic differentiation. (A1–A2) immunolabeling of undifferentiated myoblasts (T0), (B1–B2) C2C12 at one day of differentiation (T1), (C1–C4) at intermediate step of differentiation (T5), (D1–D4) at late stage of differentiation (T10). The second column of microphotographs (Bars: 0.1 μm) shows a higher magnification of area marked by square in the first column (Bars: 1 μm). At T0 and T1, the cell appeared as single and elongated myoblasts; at T5 and T10, both elongated and single myoblasts (C1–C2; D1–D2) and myotubes (C3–C4; D3–D4) were present. HC = heterochromatin, IG = interchromatin granules (arrowheads)

Immuno Electron Microscopy (IEM) evidenced that in the cytoplasm TNPO3 labeling appeared diffused within the cytosol and very weak on the cytoplasmic organelles (Fig. 3, first column). In the nucleus most of TNPO3 labeling occurred at the interchromatin domains close to interchromatin granules (IG), these latter showing only a weak signal (Fig. 3, second column). Few gold particles were present at the boundary of heterochromatin and nucleoli appeared weakly labeled. Undifferentiated myoblasts at T0 and T1 were intensely labeled (Fig. 3 A1–A2; B1–B2) while at T5 and T10 they showed few gold particles (Fig. 3 C1–C2; D1–D2); as an opposite, at T5 and T10, TNPO3 labeling increased both in the nucleus and in the cytoplasm of differentiated myotubes (Fig. 3 C3–C4; D3–D4), following the findings observed at confocal microscope.

### TNPO3 and SRSF1 localization during myogenic differentiation

**Fig. 4:**
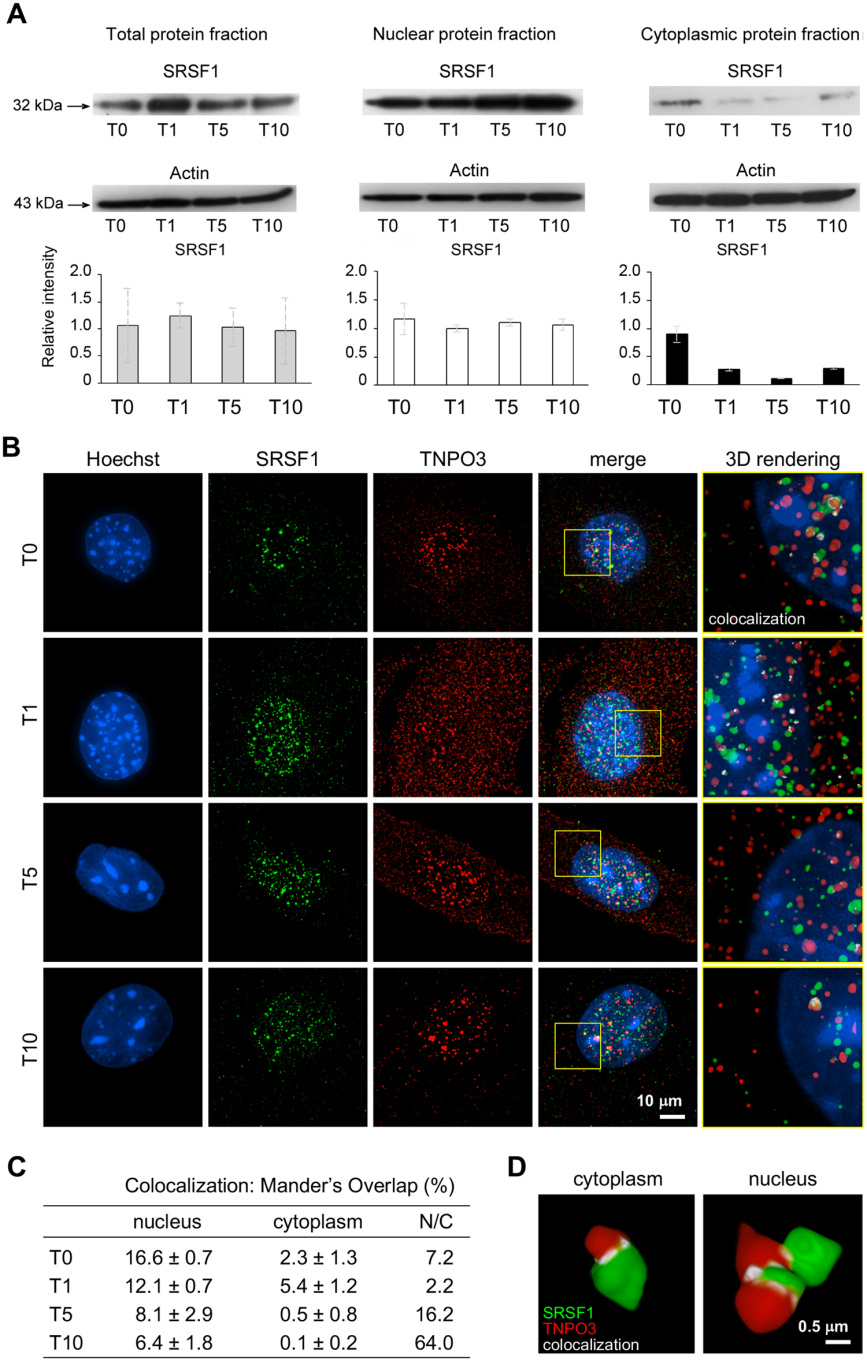
Super resolution microscopy for analysis of SRSF1 and TNPO3 interaction and quantification of colocalized fluorescent signal. (**a**) Western blotting for SRSF1 in total, nuclear and cytoplasmic protein fractions. The bands were quantitated by calculating the relative quantities of SRSF1 normalized to Actin. Data are representative of three experiments and expressed as mean ± SD. (**b**) IF double staining for TNPO3 (in red) and SRSF1 (in green) observed through a structured illumination microscope (SIM). Nuclei are counterstained with Hoechst (first column, in blue). In the fourth column merge of TNPO3 and SRSF1 fluorescent signals and in the fifth column the 3D rendering of the area marked by square; the colocalization of TNPO3 and SRSF1 is merged in white. (**c**) Colocalization has been quantified using Mander’s Overlap coefficient and is reported in table; N/C column refers to the ratio among data of colocalization in nucleus and in cytoplasm. (**d**) A detail of three-dimensional cluster analysis of colocalization (in white) for SRSF1 (in green) and TNPO3 (in red) at T5; TNPO3 globular volume is 2–3 times greater in the nucleus than in the cytoplasm

During myogenesis the expression levels of TNPO3 have been compared with the expression and localization of its cargo protein, the splicing factor SRSF1. WB analyses showed that SRSF1 expression did not change significantly during differentiation and looking to the nuclear and cytoplasmic fractions separately, SRSF1 was highly expressed in the nucleus and almost null in the cytoplasm; in addition, there were no significant differences in SRSF1 expression between the different steps of differentiation, suggesting that SRSF1 was not influenced by myogenesis (Fig. 4a).

Localization of both SRSF1 and TNPO3 has been investigated through structured illumination microscopy (SIM), which permits to observe fluorescent samples at resolutions below the limit the diffraction of light imposes by optical microscopy (85–100 nm). In undifferentiated C2C12 (T0) the expression of SRSF1 was mainly localized in the nucleus, increased at T1 and decreased at T10. Instead, TNPO3 was mainly expressed in the nucleus at T0 and achieved a similar distribution between nucleus and cytoplasm at T1. At T5 the expression of TNPO3 in the cytoplasm decreased up to T10, while in the nucleus it appeared strongly clustered (Fig. 4b). The colocalization analysis was imaged (Fig. 4b right column, merged in white) and quantified by using Mander’s Overlap coefficient (Fig. 4c). The data indicated that the colocalization between SRSF1 and TNPO3 in myoblasts was present mainly in the nucleus at T0, increased in the cytoplasm at T1 and it was almost exclusively in the nucleus of differentiated myotubes at T5 and T10, as evidenced by a ratio comparing colocalization in nucleus and cytoplasm (Fig. 4c). The three-dimensional rendering analysis of SRSF1 and TNPO3 showed regions containing highly colocalized signals (merged in white) and the analysis of colocalized signals at T5 indicated that the TNPO3 globular volume was 2–3 times greater in the nucleus than in the cytoplasm (nucleus: 1.48 ± 0.12 μm^3^; cytoplasm: 0.22 ± 0.04 μm^3^) (Fig. 4d).

### Myogenic differentiation and microRNAs analysis

**Fig. 5:**
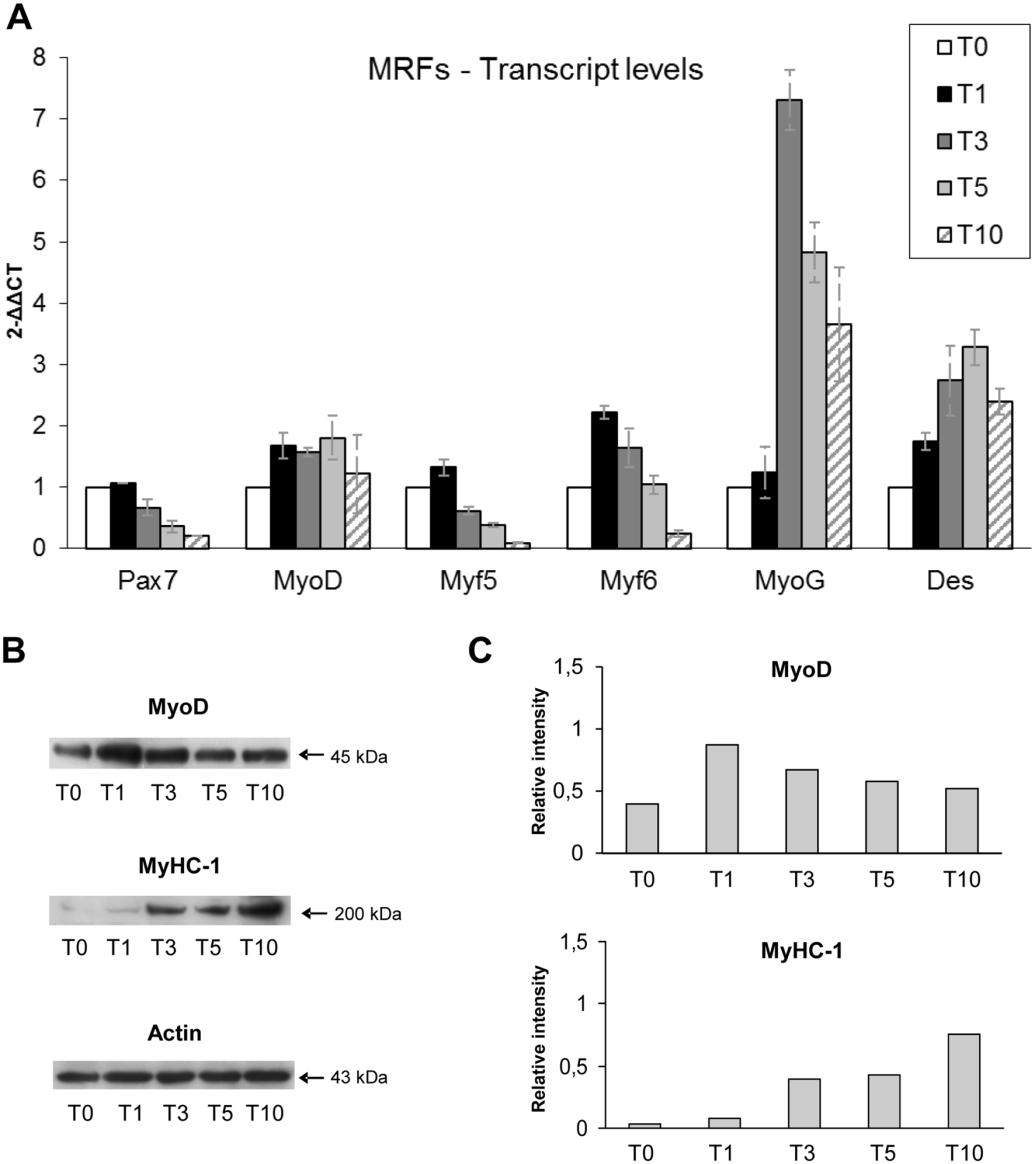
Investigation of myogenic regulatory factors (MRFs) and muscle specific proteins in C2C12 during myogenesis. (**a**) Real-time q-PCR showing the transcripts levels of early and late MRFs; data are representative of three experiments and expressed as means ± SD. (**b**) Western blotting shows a similar expression of MyoD in undifferentiated myoblasts and in the early stage of C2C12 differentiation (T0 and T1), while it decreased in the intermediate and late stages (T3–T10). On the opposite MyHC-1 starts to be expressed from the intermediate to late stage of differentiation. (**c**) The bands were quantitated by calculating the relative quantities of MyoD and MyHC-1 normalized to Actin; data are representative of three experiments

In parallel to TNPO3 analysis we investigated and checked myogenic differentiation. Undifferentiated C2C12 (T0) showed the classical myoblast phenotype while during differentiation (from T5 to T10) they started to elongate and form multinucleated myotubes. Myogenic differentiation was confirmed by the analysis of myogenic regulatory factors (MRFs) at transcript and protein level. The trend of gene and protein expression of the investigated MRFs, which normally control differentiation of skeletal muscle cells, confirmed data from literature [20] (Fig. 5a). Myogenic differentiation of C2C12 was also assessed by investigation of some muscle specific proteins (Desmin and MyHC-1) that, as expected, started to increase or to be expressed from T5 to T10 (Fig. 5b).

**Fig. 6:**
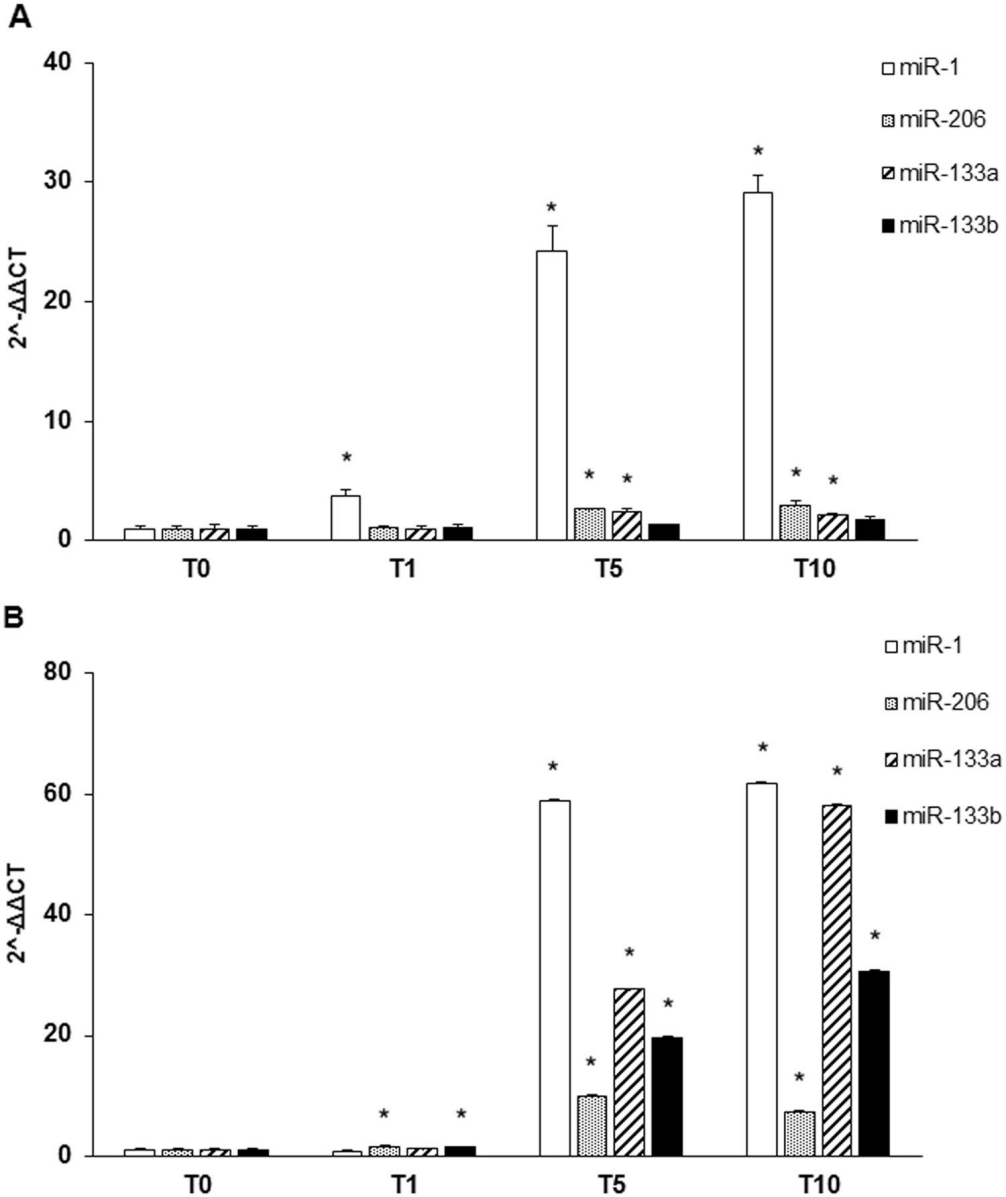
Expression of muscle specific miRNAs during myogenesis. (**a**) The expression of miR-1 in C2C12 increased immediately after 24 hrs of differentiation (T1) ant it continued to raise progressively during differentiation (T5 and T10); the trend of miR-206 was similar to that of miR-1 even if it started to increase significantly at the intermediate stage (T5). The expression of miR-133a and miR-133b was quite similar since both showed an increase in the intermediate stage (T5) remaining stable in the late stage of myogenic differentiation (T10). (**b**) The expression profile of miRNA contained in the exosomes released in the culture medium showed a peak of expression starting from the intermediate step of differentiation (T5) and was maintained till T10, while miR-206 expression showed a less marked increase at the intermediate and late stage of differentiation. Data are representative of three experiments; they are expressed as mean ± SD and the level of significance was set at *p* < 0.05

In addition to MRFs and muscle protein, we analyzed the expression of four muscle specific microRNAs (miRNAs) known as myomiRNAs (miR-1, miR-206, miR-133a and 133b) that are involved in myogenesis and muscular atrophy. During differentiation of C2C12, miR-1 showed a slight increase in T1 and a peak at T5 and T10, while miR-206 and miR-133a/b remained stable in T1 with a weak increase in T5 and T10 (Fig. 6a). Moreover, we investigated the levels of myomiRNAs released in the medium during myogenesis. MiR-1 and miR-133a/b increased from T1 with a peak of expression from T5 to T10, while miR-206 increased lightly remaining quite stable in T5 and T10 (Fig. 6b).

## Discussion

The post transcriptional gene regulation and, specifically, alternative splicing affect proteomic variability in muscle and contribute to satellite cell differentiation and myogenesis [18]. Given the importance of splicing to guarantee the specialized function of skeletal muscle, it is conceivable that alterations in the splicing mechanism might contribute to the development of large number of myopathies and muscular dystrophies [18, 28]. Alternative splicing alterations could be due to mutations located within splicing regulatory sequences or in genes encoding for splicing regulators or for factors that regulate alternative splicing decisions as well as associated proteins [29]. TNPO3 normally transports the splicing factors SRSF1 from the cytoplasm to the nucleus, so a mutation in TNPO3, such as the one described in LGMD D2 (previously LGMD1F) [30–35], could dysregulate SRSF1 localization and function, causing alterations in the alternative splicing machinery which could, in turn, affect myogenesis and the maintenance of healthy muscle.

According to these observations we analyzed the expression of TNPO3, SRSF1 and their relationship during myogenesis. As myogenic differentiation model we used C2C12, murine myoblasts derived from satellite cells whose behavior *in vitro* correspond to that of progenitor lineage [20]. C2C12 differentiation has been monitored checking the expression of specific MRFs (such as MyoD, Myf5, Myf6, MyoG), that act synergically to correctly drive muscle differentiation [21]. We analyzed also a selection of myomiRNAs, whose expression is controlled by MRFs through important feedback loops and that have an active role during myogenesis [36–41]. In detail, MyoD and insulin-like growth factor 1 (IGF-1) turn on miR-1 and miR-206 which both downregulate Pax3 and Pax7, leading to the activation of genes responsible for upregulation of Myf5 and MyoD; this positive feedback loop, at the onset of myogenic differentiation, results in cell cycle arrest and proliferation block in favor of myoblast commitment and proliferation [42, 43]. Moreover, miR-1 and miR-206 inhibit HDAC4 (histone deacetylase 4), a transcription repressor of many muscle genes among which MEF2 and MyoG, so its inhibition promotes myoblast differentiation toward myotubes [36, 40, 43]. Both miR-133a/b suppress myoblast proliferation and promote differentiation by regulating MAPK signaling and, interestingly, miR-133 expression, which is upregulated by IGF-1 via MyoG induction, produces a negative feedback loop through the suppression of the IGF-1 receptor that attenuates MyoG and results in myofibers maturation [43]. Moreover, the role of myomiRNA in muscle differentiation and regeneration has been directly demonstrated by Nakasa and collaborators showing that an injection of a mixture of miR-1, miR-133 and miR-206 in injured muscles subsequently led to muscle regeneration with an increment of MyoG, MyoD and Pax7 and prevented fibrosis [44].

Besides the involvement of myomiRNAs in muscle regeneration, alterations of circulating miRNAs have been documented in several muscular dystrophies and studying their levels could help to understand how they might influence myogenesis in the muscle of dystrophic patients [45–48]. Moreover, miR-206 have been described to significantly increase in patients affected by LGMD D2, a dominant form of LGMD due to a mutation in the TNPO3 gene; the described increase could open a new perspective for this myomiRNA as biomarker of disease severity and evolution in LGMD D2 patients [Pegoraro V et al. 2020, To be submitted].

We observed that C2C12 myoblasts normally express TNPO3, but its levels undergo quantitative variation in the nuclear and in the cytoplasmic compartments in those myoblasts that respond to myogenic stimuli by differentiating in myotubes. Investigation at confocal microscope led us to demonstrate that TNPO3 increases and is mainly present in those cells that express MyHC-1 and that can be considered differentiating myotubes. Moreover, IEM showed TNPO3 labeling in the nucleus and particularly in nuclear interchromatin domains close to IG where perichromatin fibrils, the sites where transcription and co-transcriptional splicing of mRNA occur [49], are located. It is therefore conceivable that TNPO3, involved in cytoplasmic/nucleus transport of splicing factors, appears to be localized at these sites. These data suggest an involvement of TNPO3 in the myogenic process, probably transporting some proteins that might contribute to myogenesis. Therefore, we investigated the expression of SRSF1 and its relationship with TNPO3 during myogenesis through a structured illumination microscope and we found that SRSF1 is consistently localized in the nucleus during the whole differentiation, while TNPO3 expression changes, decreasing in the cytoplasm and appearing strongly clustered in the nucleus of differentiated myotubes. What is more interesting is the analysis of colocalization between TNPO3 and SRSF1, which indicates that they are found almost exclusively in the nucleus as differentiation proceeds (T5 and T10). In particular, the quantification of the colocalization signal showed that at T10 it was significantly higher in the nucleus, up to 64-fold increase, than in the cytoplasm. Moreover, the three-dimensional cluster analysis of colocalized signals indicates that TNPO3 globular volume in the nucleus is bigger than in the cytoplasm, suggesting that TNPO3 could create dimers during translocation of cargo protein or once it is in the nucleus. The possibility that TNPO3 forms dimers has been confirmed by the evidence of dimerization at high protein concentration [1] and could help to explain the dominant negative effect observed in LGMD D2 patients, for whom sequence analysis revealed the coexistence of similar amounts of both mutated and wild type TNPO3 transcripts [30]. In conclusion, the combination of different super- and ultra-resolution imaging techniques led us to describe the behavior of TNPO3 and its interaction with SRSF1 during myogenesis, looking at nuclear and cytoplasmic compartments as well. The overall data suggest that the interaction between TNPO3 and SRSF1 and the variations in TNPO3 localization follow the myogenic process and could have a role in the proteomic network that myotubes have to build during myogenesis. These observations represent a first step that could contribute to a better understanding of the role of TNPO3 and SRFSF1 in complex mechanisms, such as myogenesis and alterations that could give rise to myopathic disorders.
